# Large area Al_2_O_3_–Au raspberry-like nanoclusters from iterative block-copolymer self-assembly†

**DOI:** 10.1039/d0ra08730k

**Published:** 2020-11-11

**Authors:** Alberto Alvarez-Fernandez, Frédéric Nallet, Philippe Fontaine, Cian Cummins, Georges Hadziioannou, Philippe Barois, Guillaume Fleury, Virginie Ponsinet

**Affiliations:** CNRS, Univ. Bordeaux, Centre de Recherche Paul Pascal, UMR 5031 115 Avenue Schweitzer 33600 Pessac France; CNRS, Univ. Bordeaux, Bordeaux INP, LCPO, UMR 5629 F-33600 Pessac France guillaume.fleury@u-bordeaux.fr; Department of Chemical Engineering, University College London Torrington Place London WC1E 7JE UK alberto.fernandez@ucl.ac.uk; Synchrotron SOLEIL L'Orme des Merisiers, Saint-Aubin-BP 48 F-91192 Gif-sur Yvette Cedex France

## Abstract

In the field of functional nanomaterials, core–satellite nanoclusters have recently elicited great interest due to their unique optoelectronic properties. However, core–satellite synthetic routes to date are hampered by delicate and multistep reaction conditions and no practical method has been reported for the ordering of these structures onto a surface monolayer. Herein we show a reproducible and simplified thin film process to fabricate bimetallic raspberry nanoclusters using block copolymer (BCP) lithography. The fabricated inorganic raspberry nanoclusters consisted of a ∼36 nm alumina core decorated with ∼15 nm Au satellites after infusing multilayer BCP nanopatterns. A series of cylindrical BCPs with different molecular weights allowed us to dial in specific nanodot periodicities (from 30 to 80 nm). Highly ordered BCP nanopatterns were then selectively infiltrated with alumina and Au species to develop multi-level bimetallic raspberry features. Microscopy and X-ray reflectivity analysis were used at each fabrication step to gain further mechanistic insights and understand the infiltration process. Furthermore, grazing-incidence small-angle X-ray scattering studies of infiltrated films confirmed the excellent order and vertical orientation over wafer scale areas of Al_2_O_3_/Au raspberry nanoclusters. We believe our work demonstrates a robust strategy towards designing hybrid nanoclusters since BCP blocks can be infiltrated with various low cost salt-based precursors. The highly controlled nanocluster strategy disclosed here could have wide ranging uses, in particular for metasurface and optical based sensor applications.

## Abstract 2

Large area Al_2_O_3_–Au raspberry-like nanoclusters and other complex structures have been created by iterative block-copolymer self-assembly, paving the way to a new generation of on-demand metallic architectures.

## Graphical abstract

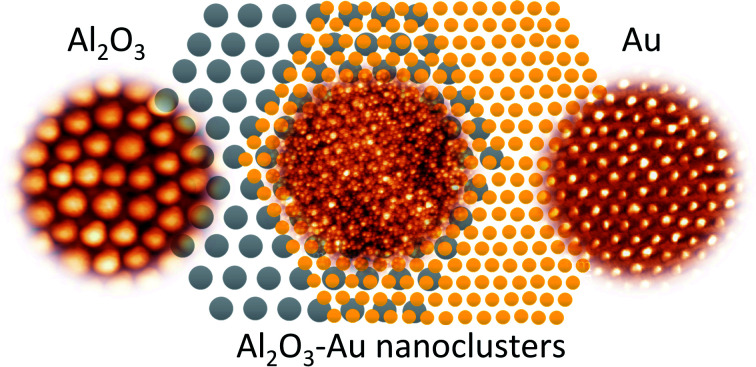

## Introduction

The appeal of nanostructured bimetallic surfaces is expanding in many fields of research and technology, due to their interesting specific properties (catalytic, magnetic, ferroelectric, mechanical, optical and electronic). Many applications are now possible based on bimetallic (or trimetallic) features such as analytic spectroscopy,^1–3^ data storage,^4^ optics and electromagnetism control,^5,6^ or control of wetting and antifouling.^7^ The scientific drive for diversity and expanded functionalities leads to an ever-revived search for new fabrication methodologies, giving access to nanostructures including metal, inorganic or hybrid materials, with controlled morphologies and characteristic sizes. Self-assembly inducing spontaneous organization in soft condensed matter is undoubtedly a promising tool.^8,9^ Some self-assembled structures, which spontaneously organize due to intermolecular interactions of energy close to thermal energy *kT*, present characteristic sizes at the nanoscale and variable degrees of order, including liquid crystals and block copolymers (BCPs). BCP nanopatterning is only one of the many opportunities offered by macromolecules at interfaces or other self-assembled materials, but it indisputably presents many advantages^10^ compared to alternative methods for the production of nanopatterned surfaces, including the tunability of domain size and morphology, the ease of processing on many types of surfaces and the possibility to smarten the systems up with chemical functionalities.

BCPs in their simplest form *i.e.* a diblock copolymer A–B (di-BCP), are comprised of two distinct polymer chains that are covalently joined. BCP self-assembly is a well-known strategy to design and create ordered structures in thin films with tunable size and morphology through the control over the BCP macromolecular parameters. di-BCP material characteristics such as the relative volume fraction of the blocks (*ϕ*), the degree of polymerization (N), the Flory–Huggins interaction parameter (*χ*_AB_) as well as the thin film process (*i.e.*, deposition and annealing conditions) enable the fine control of morphology and feature sizes.^11,12^ Subsequently, the production of large scale arrays of nanoparticles (NP) has been demonstrated by the selective hybridization with metallic or dielectric species of the nanostructured BCP scaffolds.^13–17^ However, the energy minimization during self-assembly process for common di-BCPs limits the number of accessible NPs patterns such as lines or dots.^11,12^ Multi-BCPs represent a possible alternative, giving access to many more morphologies, however accessing multi-BCPs relies on complex synthesis.^18,19^ Recently, pioneering works from Rahman *et al.*^20^ have proposed that this bottleneck can be overcome using a methodology based on the iterative assembly of BCP layers. Indeed, multilayered BCP self-assembly was used to produce a library of three-dimensional structures that are absent from the native BCP phase diagram, *i.e.* spheres-on-lines or lines-on-lines, paving the way to the production of on-demand 3D structures from BCP self-assembly.^21–23^ This process, applied with creativity and ingenuity, will give access to a wide array of original final morphologies. It contributes to the other appealing characteristics of BCPs outlined above. The transfer of the morphology to materials able to provide solid-state properties (*e.g.* magnetism, plasmonics or catalysis), requires another clever processing step. Patterning of metals has been extensively reported^24–26^ and infiltration of oxides is increasingly studied^27^ and has reached a level of maturity to understand growth kinetics and thermodynamics.^28,29^ However, hybrid nanoclusters, combining more than one material, remain a challenge.^30^ Core–shell colloids have been proposed to tackle the manufacturing of hybrid nanoclusters,^31–33^ but reports on combining multiple materials and complex structure are scarce due to a lack of reproducible fabrication methodologies.

Core–satellite raspberry-like nanocluster architectures, in which satellite nanoparticles surround a central core nanoparticle are recently of great interest due to their unique optoelectronic properties.^34^ Designing core–satellite raspberry-like nanoclusters has impacted a broad range of application areas, such as surface-enhanced fluorescence,^35,36^ Raman scattering,^37–40^ optical magnetism,^41^ drug delivery^42^ and sensing.^43–45^ Bottom-up approaches based on colloidal chemistry have been revealed as possible strategies in the synthesis of such objects.^46^ However, tedious chemical pathways associated to complex nanofabrication processes significantly hinder both scalability and development. Furthermore, even if the self-assembly of these complex structures into different 3D structures has been reported through different processes, *i.e.* microfluidics,^47^ DNA interactions^48^ or non-specific electrostatic force,^49^ the ordering of such structures into a surface monolayer still constitutes a scientific challenge.^50^ Thus, simplified routes, *i.e.* methodologies that are based on rapid and controllable processing, are needed to overcome the significant roadblocks of colloidal chemistry strategies. In this regard, the inherent characteristics of BCPs provide a possible solution to forming raspberry-like nanosurfaces.

In this work, we demonstrate the robust fabrication of core–satellite Al_2_O_3_/Au raspberry nanoclusters using multi-layered self-assembled BCP films. This methodology leads to well-defined arrays of bimetallic raspberry nanoclusters obtained from simple processing steps using conventional wet chemistry practices.^46,47^ Different molecular weight poly(styrene)-*block*-poly(2-vinylpyridine) (PS-*b*-P2VP) and poly(styrene)-*block*-poly(4-vinylpyridine) (PS-*b*-P4VP) were synthetized and self-assembled into hexagonal out-of-plane cylindrical arrays with different structural dimensions. Next, metallic-dielectric hybrid NPs were generated using the sequential self-assembly of the BCP layers selectively impregnated with different metallic-dielectric combinations. Large scale ordered monolayers have been obtained for the first time using a one-step process. Moreover, the approach described here allows control over the shape, size and density of particles owing to the flexibility of BCP nanopatterning and metal infiltration methods that can be expanded to create a library of bi- or tri-metallic species.

## Experimental

### Materials

*Sec*-Butyllithium (*sec*-BuLi, 1.2 M in cyclohexane), dibutyl magnesium (1 M in heptane) and calcium hydride (CaH_2_) were purchased from Sigma Aldrich and used as received. Anhydrous lithium chloride was purchased from Fisher Scientific and used as received. Styrene (S) from Sigma-Aldrich was first distilled over CaH_2_ and then stirred over dibutyl magnesium for 2 hours. 2 and 4-Vinylpyridine (2VP and 4VP, Sigma Aldrich) as well as 1,1-diphenylethylene were distilled twice over CaH_2_. Tetrahydrofuran (THF, Sigma-Aldrich), dried over a Braun MB-SPS-800 solvent purification system, was additionally distilled over sodium benzophenone ketyl prior to use. Acid tetrachloroauric (HAuCl_4_) (99.999% trace metals basis) and propylene glycol monomethyl ether acetate (PGMEA) (Reagent Plus, ≥99.5%) were purchased from Sigma-Aldrich and Merck respectively, and used without further purification. Silicon wafers (100) were purchased from Si-Mat Silicon Materials and cut to appropriate dimensions (approximately 1 × 1 cm^2^).

### BCPs synthesis

The PS-*b*-P2VP (*M*_*n*_ (PS) = 150.0 kg mol^−1^, *M*_*n*_ (P2VP) = 32.0 kg mol^−1^) and PS-*b*-P4VP (*M*_*n*_ (PS) = 14.7 kg mol^−1^, *M*_*n*_ (P4VP) = 6.3 kg mol^−1^) BCPs were synthesized by living anionic polymerization according to the standard procedures reported in the literature.^51,52^ In a 500 mL flamed dried round flask equipped with magnetic stirrer, THF (400 mL) was introduced. The solution was cooled to −78 °C before the sequential addition of *sec*-BuLi and styrene. The reaction mixture was stirred for 30 min and the living polystyryl lithium anions were end-capped with 1,1-diphenylethylene. VP monomers were added after 30 min and the reaction mixture was kept stirring for 30 min. Finally, the reaction was terminated by the addition of degassed methanol, concentrated, precipitated in cyclohexane and dried under vacuum at 35 °C. The different BCPs were characterized by ^1^H NMR (*δ* (ppm), 400 MHz, THF-d_8_), and size exclusion chromatography (SEC) in THF using the universal calibration technique.

### PS-*b*-P2VP BCP self-assembly

Hexagonal out-of-plane cylindrical arrays formed from the BCP self-assembly in thin films were produced by spin coating a 2.5 wt% solution of PS_150k_-*b*-P2VP_32k_ (BCP1) in toluene and a 0.5 wt% solution of PS_14.7k_-*b*-P4VP_6.3k_ (BCP2) in PGMEA respectively, onto bare silicon wafers (4000 rpm, 30 s). While BCP2 samples were used as-spun, a subsequent solvent vapour annealing (SVA) treatment in THF for 16 hours was used to improve the BCP ordering for BCP1.

Out-of-plane PS-*b*-P2VP lamellae were obtained directly by spin-coating of a 1 wt% solution of PS_102k_-*b*-P2VP_97k_ (BCP3) in PGMEA onto the modified Si wafers.^15^

### Atomic layer deposition

Sequential infiltration synthesis (SIS) was performed using an ALD (Ultratech SAVANNAH G2) tool in exposure mode.^53^ This mode allows the SIS of Al_2_O_3_ dots in the VP domains using an alternating exposure of the BCP thin films to trimethylaluminum (TMA) and deionized water at 85 °C with a N_2_ purge after each exposition step. The exposure and purge times used in this study were 60 s and 300 s, respectively, and ten SIS cycles (TMA/purge/H_2_O/purge) were performed to obtain the Al_2_O_3_ dots.

### Selective Au and Pt impregnation

1 wt% solutions of HAuCl_4_ and H_2_PtCl_6_ respectively in Mili-Q water were used to impregnate the BCP films by immersion of the sample in the solution for 30 min. The samples were exposed to an O_2_ RIE plasma treatment in a PE-100 chamber (plasma conditions: 60 W, 10 sccm O_2_, 60 s) in order to etch the polymer scaffold and reduce the gold salts to metallic Au and Pt respectively.^15^

### AFM characterization

AFM (Dimension FastScan, Bruker) was used in tapping mode to characterize the surface morphology of the different films. Silicon cantilevers (FastScan-A) with a nominal tip radius of 5 nm were used. The resonance frequency of the cantilevers was about 1.25 kHz.

### GISAXS measurements

Grazing-incidence small-angle X-ray scattering (GISAXS) experiments were performed on SIRIUS (Soft Interfaces and Resonant Investigation on Undulator Source) station at the SOLEIL synchrotron in Gif-sur-Yvette (France) (8 keV).^54^ The incidence angle was set in the range of 0.12°–0.19°, which is between the critical angle of the BCP film and the silicon substrate. The beam illuminates the samples with a typical footprint of 150 mm^2^. 2D scattering patterns were collected with a PILATUS 1 M Dectris detector with a vertical beam stop in front of the detector's window. The sample-to-detector distance was set to 4459 mm. The beam center position, the sample-to-detector distance and the resulting angular range were calibrated using a silver behenate standard sample. GISAXS patterns were reduced using the FitGISAXS software.^55^

### Reflectivity measurements

The reflectivity measurements were carried out at Centre de recherche Paul-Pascal, using a custom-made instrument. X-rays were produced by a Rigaku MM007_HF rotating copper-anode generator (*λ* = 0.154 nm) equipped with a multilayer collimating monochromator from Osmic. The samples were mounted vertically on a three-circle Huber goniometer and the reflected signal was collected with a Peltier-cooled solid-state detector XR-100CR from Amptek Inc (residual noise = 10^−2^ cnts per s). The beam at sample position was *h*0.2 × *v*2 mm^2^ and the resolution along the (horizontal) normal to the substrate was Δ*q*_*z*_ = 0.018 nm^−1^ FWHM. The data were analysed by the GenX 2.4.10 software,^56^ available online at https://genx.sourceforge.io/.

## Results and discussion

**Fig. 1:**
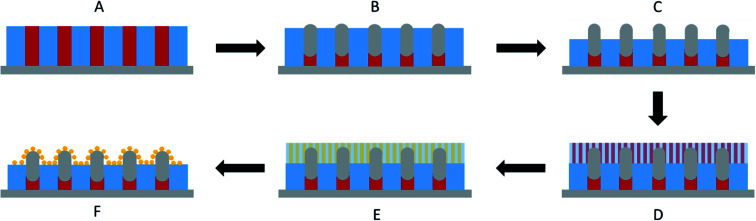
Schematic representation of the raspberry nanoclusters fabrication: (A) formation of an out-of-plane cylindrical structure obtained from BCP1 self-assembly. (B) SIS of Al_2_O_3_ by ALD into the cylindrical PVP domains. (C) Controlled removal of 30 nm polymer film by UV/O_3_ etching. (D) Formation of a second out-of-plane cylindrical structure obtained from BCP2 self-assembly on top of the first layer. (E) Au metal impregnation into the PVP domains followed by (F) Au metal reduction by RIE plasma.

Ordered monolayer distributions of raspberry-like bimetallic nanoparticles were produced by the sequential multi-layered self-assembly of PS_150k_-*b*-P2VP_32k_ (BCP1) and PS_14.7k_-*b*-P4VP_6.3k_ (BCP2), following the methodology sketched in Fig. 1.

**Fig. 2:**
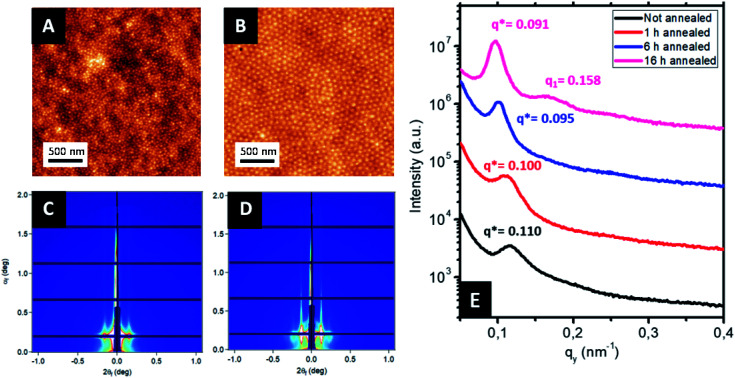
AFM topographical images of (A) as-cast BCP1 layer and (B) self-assembled BCP1 layer after 16 h SVA. GISAXS patterns of BCP1 films at different solvent vapour annealing (SVA) time: (C) as-cast and (D) 16 h. (E) Corresponding GISAXS line-cut along *q*_*y*_ integrated around the Yoneda band.

In a first step, a 2.5 wt% solution of BCP1 in toluene was spin-coated onto bare silicon wafers resulting in a BCP layer of 90 nm thickness. Considering the low mobility of the high molecular BCP1 chains, an ill-defined self-assembled structure is obtained as shown on the AFM topographical image (see Fig. 2A).^57–59^ Therefore, a subsequent solvent vapour annealing (SVA) treatment using THF was used to improve the BCP ordering. GISAXS experiments were performed at different SVA durations to elucidate and to define an optimal SVA BCP ordering process window. The GISAXS pattern of the as-cast BCP1 sample (Fig. 2C) presents a unique broad Bragg rod, revealing the out-of-plane orientation of the BCP structure. The lack of higher order reflections suggests a poor in-plane ordering of the self-assembled structure obtained directly after casting corroborating the AFM characterization. For solvent-annealed BCP1 films, higher order Bragg rods are evident on the GISAXS patterns, indicating an improved translational order of the BCP hexagonal structure (see ESI, Fig. S1†). After 16 h of SVA, the first order Bragg rod is positioned at q* = 0.091 nm^−1^ (corresponding to a center-to-center distance of *d*_C–C_ = 80 nm between cylinders in accordance with the AFM characterization) and higher order Bragg rods at 
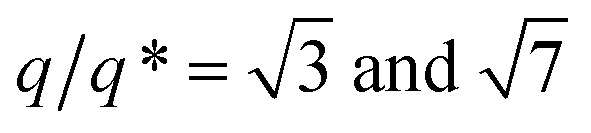
 are clearly visible (see Fig. 2D) confirming the hexagonal packing. Using the GISAXS intensity curves of the first order Bragg rod (see Fig. 2E), the full width at half-maximum (FWHM) was estimated for the different annealing time in order to extract the correlation length, *ξ*, (typical length scale over which the self-assembled morphology has conserved orientational and positional order) using Scherrer analysis.^60^ For longer SVA duration, larger *ξ* were observed (see ESI, Fig. S2†). Optimal results were obtained after 16 h of SVA, with a *ξ* of ≈350 nm. These results are further confirmed by the nanostructured BCP1 film AFM image recorded after 16 h of SVA which shows a well-ordered hexagonal out-of-plane cylindrical arrays of P2VP domains in a PS matrix (Fig. 2B).

**Fig. 3:**
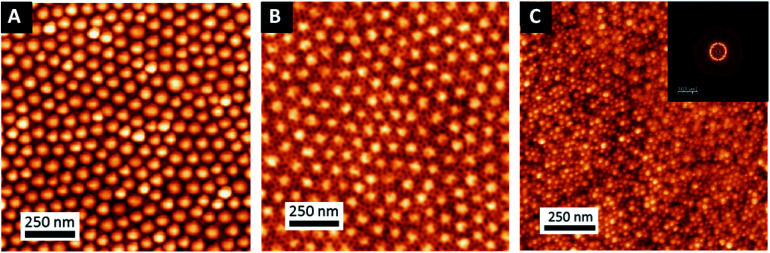
AFM topographical images of the different steps of the process to obtain bimetallic raspberry-like nanoclusters: (A) alumina dots obtained after SIS and 30 min of treatment with UV/O_3_. (B) Self-assembled BCP2 layer on top of the Al_2_O_3_ dots generated from the SIS of the self-assembled BCP1 film and (C) hexagonal ordered raspberry-like nanoclusters (Au@Al_2_O_3_) monolayers obtained by iterative self-assembly. Inset in (C) correspond to the FFT of the AFM image. Two different periodicities are clearly identified, corresponding to the Al_2_O_3_ dots and Au NPs respectively.

The sequential infiltration synthesis (SIS) of Al_2_O_3_ within the cylindrical VP domains was subsequently carried out to replicate the self-assembled BCP structure into hexagonally packed Al_2_O_3_ dots. After 10 SIS cycles, Al_2_O_3_ was selectively introduced into the P2VP domains^61–64^ and a subsequent etching step using UV/O_3_ in order to remove around 30 nm of the BCP film thickness revealed the Al_2_O_3_ dots localized at the BCP/air interface (see Fig. 3A). Accordingly, this step introduced a topographical field to guide the self-assembled BCP2 structure formed through the spin coating of a 30 nm thick layer as shown in Fig. 3B. By a subsequent immersion of the multi-layered structure into an aqueous metallic salt solution (HAuCl_4_, 1 wt% in H_2_O) for 30 minutes, the gold salts were selectively incorporated into the P4VP domains. In a final step, the samples were exposed to an O_2_ RIE plasma (30 s, 10 sccm and 60 W) in order to trigger the reduction of the gold salts into metallic gold while removing the BCP2 scaffold.^15^ The AFM topographical view of the final structure is presented in Fig. 3C and consists in hexagonally packed Al_2_O_3_ dots (diameter around 36 nm) decorated by smaller gold dots (diameter around 15 nm). SEM image confirms the ordered structure created with distinct Au dots atop the larger diameter Al_2_O_3_ dots (see ESI, Fig. S3†).

**Fig. 4:**
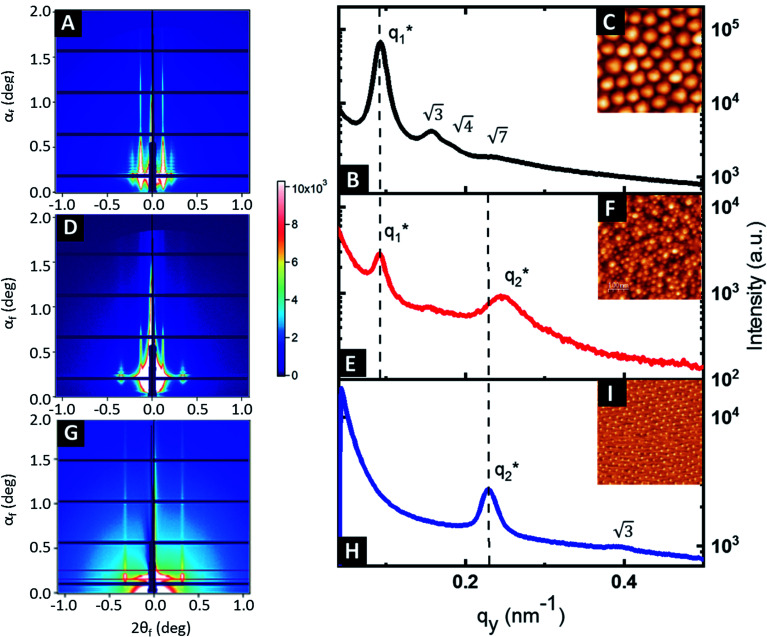
GISAXS patterns of (A) the Al_2_O_3_ dots formed by the infiltration of BCP1, (D) the bimetallic raspberry-like nanoclusters obtained by the combination of the small Au^0^ NPs (∼15 nm diameter) on top of the Al_2_O_3_ dots (∼36 nm diameter) and (G) Au^0^ NPs obtained using a nanostructured BCP2 film as a template. (B), (E) and (H) correspond to the GISAXS line-cuts along *q*_*y*_ integrated around the Yoneda band (insets: AFM topographical images of the corresponding nanostructures (C), (F) and (I)).

GISAXS experiments were performed at each step of the process to follow the iterative self-assembly process and verify the out-of-plane orientation of features and their degree of ordering. Fig. 4A shows the GISAXS pattern obtained for the samples composed of Al_2_O_3_ dots made by SIS from the nanostructured BCP1 film. A sequence of Bragg rods 
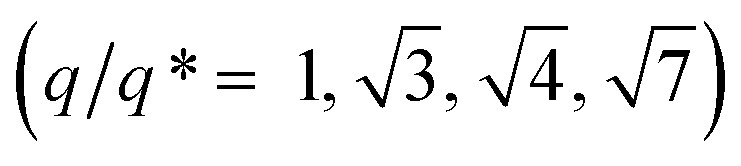
 consistent with an out-of-plane hexagonal packing of the P2VP cylindrical domains is retrieved and the centre-to-centre distance 
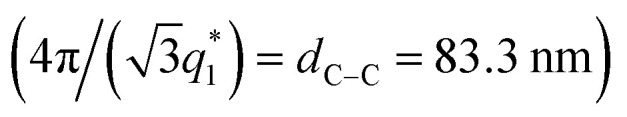
 extracted from the GISAXS pattern is in good agreement with the AFM characterization (*i.e.* 79.4 nm, see ESI, Fig. S4†). The GISAXS pattern obtained after the deposition of the BCP2 layer on top of the Al_2_O_3_ array differs drastically as shown in Fig. 4D. An additional intense Bragg rod appears at *q* = 0.24 nm^−1^ while the GISAXS pattern line-cut along *q*_*y*_ integrated around the Yoneda band confirms that the morphological characteristics of the Al_2_O_3_ array remains unchanged (Fig. 4B and E). The additional Bragg rod observed at *q* = 0.24 nm^−1^ is attributed to the Au arrays formed on top of the Al_2_O_3_ layer even if the position of the Bragg rod is modified as regards to the neat BCP2 nanostructured film due to the topography swelling induced by the Al_2_O_3_ array (see Fig. 4G and H). It is noteworthy that the apparent broadening of this particular Bragg rod hints to a poor translational order of the Au^0^ dots array confirmed by the absence of higher order Bragg rods at higher *q* values. Nevertheless, the GISAXS data are in excellent agreement with the formation of ordered arrays of bimetallic nanoclusters decorating the substrate surface over a large scale through this iterative self-assembly strategy.

**Fig. 5:**
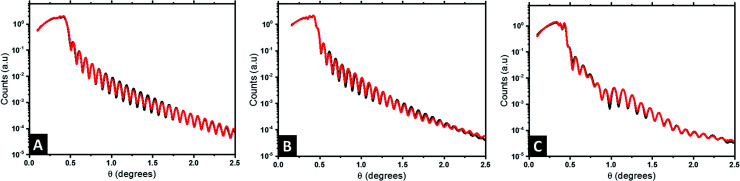
XRR reflectivity data (black line) obtained for (A) nanostructured BCP1 film, (B) Al_2_O_3_ dots array and (C) the final Au@Al_2_O_3_ bimetallic raspberry-like nanoclusters. Red lines correspond with fitting obtained by GenX.

We have additionally studied the internal stratification between the components (*i.e.*, perpendicular to the substrate) using X-ray reflectivity (XRR). It constitutes one of the most efficient techniques to characterize the structure of thin films with high sensitivity, as it offers a high spatial resolution over large penetration depths and allows us to further corroborate the large scale nature of the Au@Al_2_O_3_ raspberry features formed.^65^Fig. 5 presents the reflectivity curves obtained at different steps of the fabrication process: neat BCP1 nanostructured film (Fig. 5A), Al_2_O_3_ dots array (Fig. 5B) and the final Au@Al_2_O_3_ bimetallic raspberry-like nanoclusters (Fig. 5C). The reflectivity fringes obtained for the BCP1 film (Fig. 5A) suggest a homogeneous film of low roughness while more complex structures are detected for the Al_2_O_3_ dots array and the final bimetallic raspberry-like nanoclusters since some destructive interferences can be observed.

In order to gain some insights on the internal structure, different structures (taking into account geometrical data derived from the AFM and GISAXS characterizations) were created using the GeniX software to model the samples by fitting with the experimental results.^56^ Theoretical values of SLD for the different elements were calculated using the procedure presented in the ESI,† and are listed in Table 1. SLD values are expressed in Å^−3^, obtained by dividing the SLD value by the Thomson radius (*r*_e_).

**Table 1:** SLD theoretical values for the materials of interest in this study

Material	Formula	Density	SLD (Å^−2^)	SLD/*r*_e_ (Å^−3^)
Silicon	Si	2.3	2.0 × 10^−5^ − *i*4.6 × 10^−7^	0.71 − *i*1.6 × 10^−2^
BCP	C_15_H_15_N	1.02	9.3 × 10^−6^ − *i*1.3 × 10^−8^	0.33 − *i*4.6 × 10^−4^
Gold	Au	19.6	1.25 × 10^−4^ − *i*1.3 × 10^−5^	4.44 − *i*0.46
Alumina	Al_2_O_3_	3.95	3.25 × 10^−5^ − *i*3.8 × 10^−7^	1.15 − *i*1.3 × 10^−2^

**Fig. 6:**
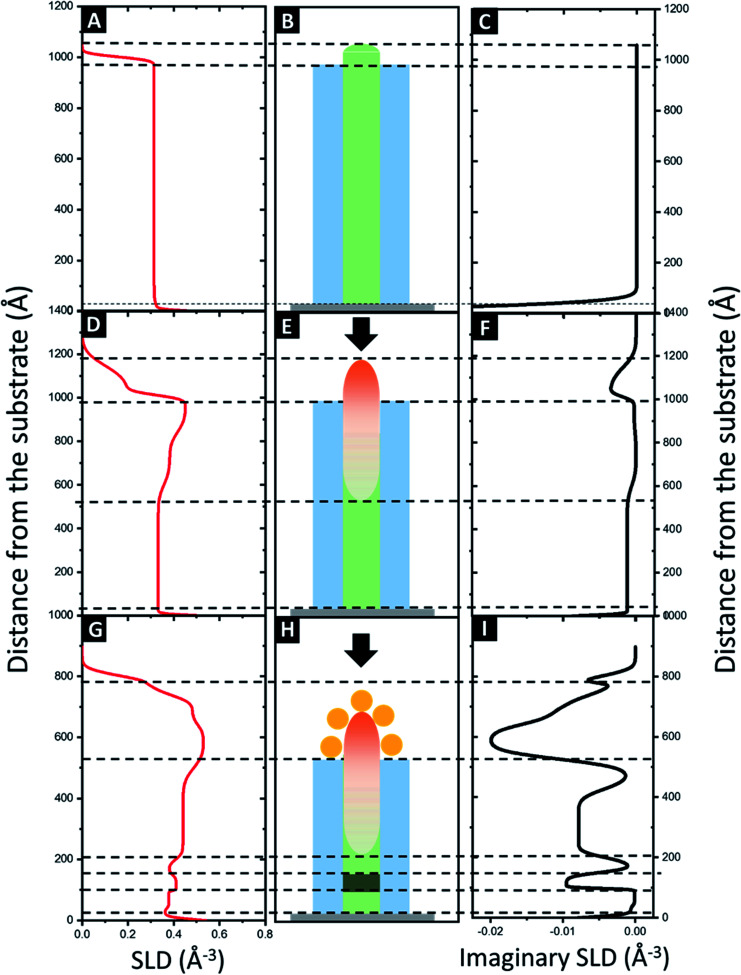
SLD profile (left) and imaginary SLD profiles (left: real part; right: imaginary part) obtained after fitting the experimental XRR data for: (A–C) neat BCP1 film, (D–F) BCP1 layer impregnated with Al_2_O_3_ and (G–I) bimetallic raspberry-like nanoclusters consisting in a Al_2_O_3_ core decorated with Au satellites. Graphical representations of the structures (B, E and H) are also presented to help the data interpretation.

In case of the neat BCP1 layer, the best fit with a figure of merit (FOM) of 4.26 × 10^−2^ (black data and red fit lines in Fig. 5A, respectively) correspond to one homogeneous layer with some roughness at the surface. The scattering length density (SLD) profile was then extracted as a function of the height *z* (Fig. 6A). For negative *z*, a SLD value of 0.71 Å^−3^ is obtained in good agreement with the theoretical value of Si. From *z* = 0, the SLD rapidly decreases to a uniform SLD value of 0.31 Å^−3^. Since the theoretical SLD value expected for the polymer is 0.33 Å^−3^, the experimental value is consistent with a homogeneous BCP film. At the upper surface of the film, the SLD decreases with a certain interface thickness, due to the topography of the BCP1 layer (see Fig. 6B). Such behaviour can indeed be related to the quasi-hemispherical shape of the P2VP cylinders protruding from the PS matrix. The thickness of the sample is found to be 103 nm, including the native SiO_2_ layer between the silicon and the polymer. The fabrication process of the nanoclusters continues with the incorporation of Al_2_O_3_ into the P2VP domains by ALD. In this case, the model that fits best the XRR data is more complicated, as it consists of four different layers. The FOM of the fit is 5.68 × 10^−2^ showing that the proposed model matches very well the experimental results (Fig. 5B). The SLD profile obtained is therefore more complex (Fig. 6D). First, like in the previous case, a high SLD value of 0.71 is obtained at *z* = 0, which is assigned to the silicon substrate. Then the SLD value decrease until 0.33 Å^−3^ (expected value for a polymer film). The following layer has a higher SLD (0.39 Å^−3^), which we interpret as the introduction of Al_2_O_3_, since the presence of the Al_2_O_3_ is expected to lead to an increase of the electron density of the film. It is noteworthy that the incorporation of Al_2_O_3_ by SIS does not occur homogeneously in the whole cylinder height (see Fig. 6E), the sample has a thickness around 100 nm and the alumina penetration is found to be 50 nm deep into the polymer film, in agreement with previous studies where homogeneous distributions of the alumina precursors have been only observed in thinner samples (≈30 nm thick).^66^ This is clearly observed in the SLD profile, which increases progressively from around 50 nm from the surface of the substrate until reaching a maximum value of 0.46 Å^−3^ at 100 nm. Thereby, the volume fraction, *Φ*_Al_2_O_3__, of the Al_2_O_3_ though the film is not constant and can be estimated with the following expression.

1 SLD_composite_ = SLD_BCP_*Φ*_BCP_ + SLD_Al_2_O_3__*Φ*_Al_2_O_3__

where SLD_BCP_ = 0.31 Å^−3^, SLD_Al_2_O_3__ = 1.5 Å^−3^ and *Φ*_BCP_ + *Φ*_Al_2_O_3__ = 1. A volume fraction of 7% of Al_2_O_3_ is calculated at 70 nm thickness, increasing to 13% close to the film surface. In the next step, the sample was exposed to a UV/O_3_ treatment in order to remove partially the BCP layer. Then the second film (*i.e.* BCP2) was spin-coated on top of the Al_2_O_3_ array and impregnated with the gold salt. An O_2_ RIE treatment was carried out to remove this BCP2 layer and reduce the gold salt to metallic gold. Bimetallic nanostructures composed of an Al_2_O_3_ core covered by metallic Au satellites were obtained on the top of the surface. In this case, the model requires to appropriately correlate with the reflectivity data is even more complex and 12 different layers were necessary to obtain a satisfactory fit of the XRR data (Fig. 4C). The FOM obtained with this model is 3.07 × 10^−2^ and the SLD profile obtained is presented in Fig. 6G.

At *z* < 0 and *z* = 0, the silicon substrate is observed as well as a thin layer of SiO_2_ corresponding to a SLD value of 0.71 Å^−3^, in agreement with the theoretical values listed on Table 1. Between *z* = 0 and *z* = 20 nm, three thin layers with SLD values of 0.37 Å^−3^, 0.41 Å^−3^ and 0.37 Å^−3^, respectively were used to satisfactory fit the XRR data. The SLD values are higher than expected for the pristine polymer (0.33 Å^−3^), suggesting the presence of some metallic impurity. One possible explanation could be related to the presence of the residual BCP1 layer below the Al_2_O_3_ particles which is weakly impregnated by the gold salt solution at the time of the formation of the Au dots.

Above, the SLD value increases again due to the presence of the Al_2_O_3_. A constant SLD value of 0.44 Å^−3^ is observed from 22 to 44 nm, corresponding to a *Φ*_Al_2_O_3__ of 11.3%. Therefore, a densification of the Al_2_O_3_ layer is observed (from the previous 7% to the current 11.3%). This can be attributed to the partial removal of the BCP1 layer by UV/O_3_.^66,67^ A higher SLD value (0.53 Å^−3^) is observed near the surface of the film due to the presence of the gold NPs (also the imaginary part decreases to highly negative values as shown in Fig. 6I). Finally, the decrease of the SLD is consistent with the presence of Au NPs on top of the larger alumina dots, corroborating the formation Au@Al_2_O_3_ raspberry-like nanoclusters (Fig. 6H).

**Fig. 7:**
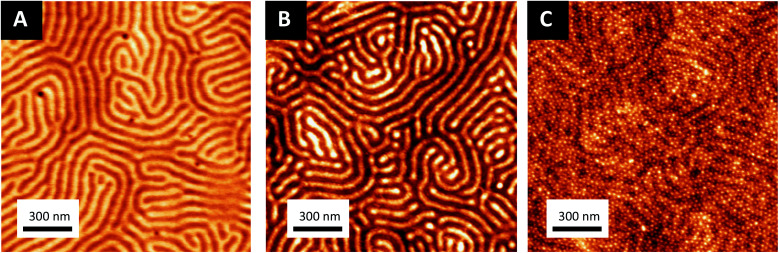
AFM topographical images of the different steps of the process to obtain decorated bimetallic nanowires (A) out-of-plane lamellar structure obtained from BCP3 self-assembly (B) Pt nanowires obtained by selectively impregnation out-of-plane BCP3 lamellar films and (C) bimetallic Au–Pt nanowires obtained by self-assembly of BCP2 on top of the Pt nanowires.

In order to generalize this strategy, and show the high versatility of this approach, another complex nanostructure was studied. In this case, a first layer of metallic platinum nanowires (Fig. 7B) was obtained after replication of an out-of-plane lamellar structure obtained from BCP3 self-assembly (see Fig. 7A). Once the platinum nanowires were obtained, a 0.5 wt% solution of BCP2 in PGMEA was spin coated on top of it. After a subsequent immersion in the gold precursor solution, a hexagonal array of gold nanodots was obtained, decorating the platinum nanowires and creating, in this case, a spheres-on lines bimetallic complex structure (Fig. 7C). These two examples show the high versatility of this strategy to produce different hybrid structures, combining not only different materials but also different morphologies giving rise to a wide variety of metallic–metallic or metallic–dielectric combinations.

## Conclusion

We have presented here a new, versatile and straightforward strategy to obtain on-demand bimetallic Au@Al_2_O_3_ raspberry-like nanoclusters using multi-layered self-assembled BCP layers. For the first time, highly ordered monolayers of an Al_2_O_3_ core with Au satellites have been obtained. Several techniques, *i.e.*, AFM, SEM, GISAXS and XRR, have been used to confirm the high degree of order of the designed structures, exemplifying the robustness and reproducibility of the Au@Al_2_O_3_ nanocluster fabrication process. Indeed, we believe a wide variety of metallic–metallic or metallic–dielectric combinations are accessible with this approach, giving rise to the possibility of exploring unique surface properties. The versatility and highly controlled nature of the nanocluster strategy disclosed here surpass traditional wet chemistry methods, and therefore could find use for wide ranging applications. For example, such bimetallic features could be applied as optical metasurfaces, catalytic active sites for material growth or label free biosensors where nanodimensional control are all-important.

## Conflicts of interest

There are no conflicts to declare.

## Supplementary Material

RA-010-D0RA08730K-s001
