# Supplementary material for: Large area Al2O3–Au raspberry-like nanoclusters from iterative block-copolymer self-assembly
Source: RSC Adv. 2020 Nov 11;10(67):41088–97. doi: 10.1039/d0ra08730k (PMC9057902; doi:10.1039/d0ra08730k)
Supplement: RA-010-D0RA08730K-s001 [file RA-010-D0RA08730K-s001.pdf]

Electronic Supplementary Material (ESI) for RSC Advances.
This journal is © The Royal Society of Chemistry 2020

Supporting information

Large area Al_2O_3-Au raspberry-like nanoclusters from iterative block-copolymer self-assembly.

Alberto Alvarez-Fernandez[a,b‡], Frédéric Nallet[a], Philippe Fontaine[c], Cian Cummins[a,b], Georges Hadziioannou[b], Philippe Barois[a], Guillaume Fleury[b*] and Virginie Ponsinet[a]*

[a] CNRS, Univ. Bordeaux, Centre de Recherche Paul Pascal, UMR 5031, 115 Avenue Schweitzer, 33600 Pessac, France

[b] Univ. Bordeaux, CNRS, Bordeaux INP, LCPO, UMR 5629, F-33600, Pessac, France
E-mail: guillaume.fleury@u-bordeaux.fr

[c] Synchrotron SOLEIL, L'Orme des Merisiers, Saint-Aubin-BP 48, F-91192 Gif-sur Yvette Cedex, France

‡ Present address: Department of Chemical Engineering, University College London, Torrington Place, London, WC1E 7JE, UK. Email: alberto.fernandez@ucl.ac.uk

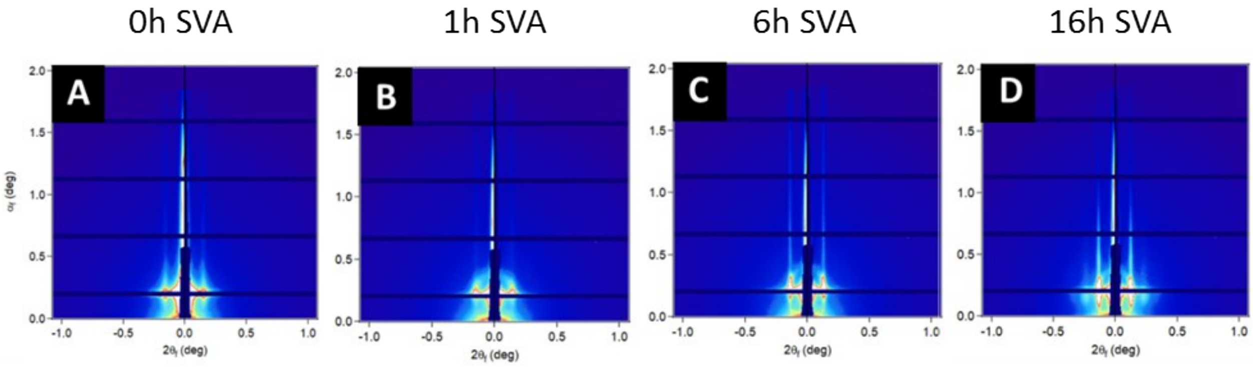

Figure S1. GISAXS patterns of BCP1 films at different solvent vapour annealing (SVA) time: (A) as-cast, (B) 1h, (C) 6h and (D) 16h.

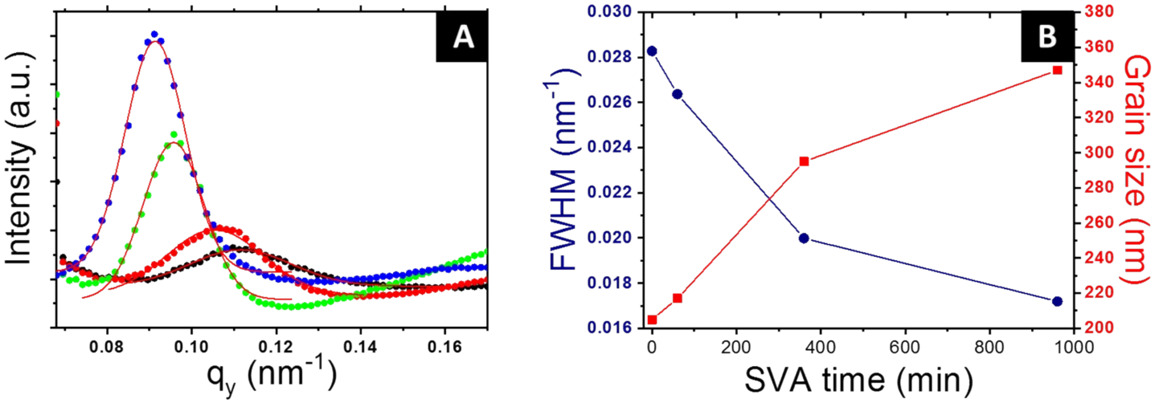

Figure S2. (A) First Bragg rod peak for the BCP1 sample at different SVA times: 0 h (black), 1 h (red), 6 h (blue) and 16 h (green), and the corresponding Gaussian fits in order to estimate the FWHM. (B) FWHM and the grain size values obtained by Scherrer analysis.[1]

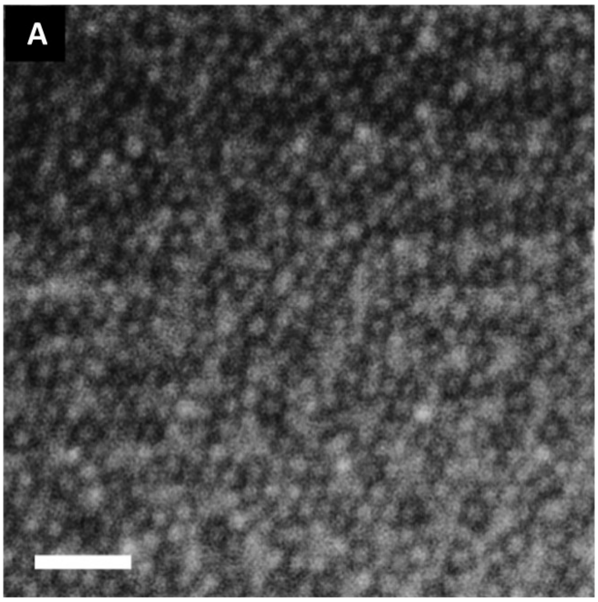

Figure S3. top-down SEM image of the hexagonal ordered raspberry-like nanoclusters ($Au@Al_2O_3$) monolayers obtained by iterative self-assembly.

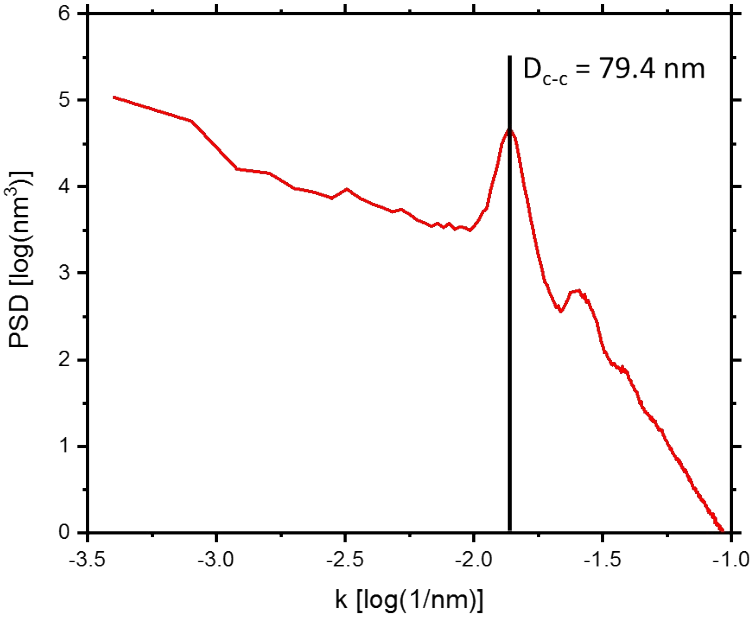

Figure S4. Power spectral density function calculated as the square of the absolute intensity value of the FFT for the Al_2O_3 dots obtained by SIS from the nanostructured BCP1 film.

Reflectivity analysis (GenX software)

Even if some information can be obtained directly from the reflectivity curve, *i.e.*, the thickness and the density of the film, it is necessary to establish a model to go further in the analysis and to extract all the information required for a better understanding of the internal structure of the film.

In an absorbent medium, the refractive index, *n*, in the wavelength range of X-rays can be defined as:

$$n = 1 - \delta - i\beta$$

With the real term δ related to the dispersion, and the imaginary part β related to the absorption. In the X-ray wavelength range, both δ and β are small quantities (about 10^{-4} to 10^{-8}). In order to fit non-homogeneous density profiles, one usual approach consists in splitting the whole thickness of the film in several independent homogeneous layers, using the GenX software.[2] δ and β are constant in each of these individual layers. The fit uses the Parrat formalism, which iterates the calculation of the transmission and reflection at each interface to obtain at the end of the process an adjusted profile of the scattering length density (SLD) over the whole film thickness. The SLD iterated by GenX is related with δ and β by the following expressions, where r_e is the Thomson radius (2.818×10^{-15}m), and λ the X-ray wavelength.

$$\delta = r_e \frac{\lambda^2}{2\pi} R(SLD)$$

$$\beta = r_e \frac{\lambda^2}{2\pi} I(SLD)$$

Theoretical SLD values calculation

SLD can be computed from the scattering lengths and material densities as:

$$SLD = \frac{\sum_{i=1}^{N} b_i}{V_m}$$

Where we sum the scattering length contributions (b_i) from the N atoms, divide by the volume, V_m, of this unit cell, that can be represented as:

$$V_m = \frac{M}{\rho N_a}$$

Where M is the molar mass, N_a is the Avogadro constant and ρ is the mass density. X-ray scattering length contribution (b_i) can be calculated from the tabulated atomic scattering factors for each atom as:

$$b_i = \frac{e^2}{4\pi\varepsilon_0 m_e c^2} f_1$$

Where f_1 is the real part of the atomic scattering factor (and can be approximated as equal to the atomic number Z), e is the charge of the electron, ε_0 is the permittivity of free space, m_e is the mass of the electron and c is the speed of light. During all this work SLD values have been expressed in $Å^{-3}$, obtained dividing the SLD value by the Thomson radius ($r_e = 2.818 \times 10^{-15}$ m).

1 D.-M. Smilgies, *J. Appl. Crystallogr.*, 2009, **42**, 1030–1034.

2 M. Björck and G. Andersson, *J. Appl. Crystallogr.*, 2007, **40**, 1174–1178.
